# Importancia del diagnóstico por imagen en el diagnóstico precoz de la mucormicosis pulmonar: a propósito de un caso

**DOI:** 10.1016/j.opresp.2021.100091

**Published:** 2021-03-24

**Authors:** Marta Porta Vilaró, Sebastián Capurro, Daniel Martínez, Ivan Vollmer

**Affiliations:** a Servicio de Radiodiagnóstico (CDIC), Hospital Clínic, Barcelona, España; b Servicio de Anatomía Patológica (CDB), Hospital Clínic, Barcelona, España

*Dear Editor:*

La mucormicosis pulmonar es una infección oportunista causada por hongos del orden *Mucorales*. Se adquiere mediante la inhalación de esporas, y su propagación está favorecida por enfermedades como la diabetes, la enfermedad renal crónica o la inmunosupresión. La enfermedad se caracteriza por angioinvasión e invasión de estructuras vecinas^1^, ^2^.

Es importante diferenciarla de la aspergilosis pulmonar, ya que ambas afectan a pacientes inmunodeprimidos. Estos pacientes reciben profilaxis contra aspergilosis, pero no contra mucorales, motivo por el cual se está observando un incremento relativo en la incidencia de mucormicosis^2^. Las 2 pueden tener características por imagen parecidas, observándose en estadios iniciales opacidades en vidrio deslustrado peribroncovasculares que progresan a nódulos o masas rodeadas por un halo en vidrio deslustrado. Un hecho diferencial es que la mucormicosis tiende a invadir los vasos y es frecuente observar áreas de necrosis pulmonar y amputación de vasos, para lo cual es especialmente útil un estudio con contraste intravenoso. Ocasionalmente, la aparición de una cavidad en el parénquima pulmonar puede ser la manifestación inicial de la enfermedad^1^, ^2^.

Presentamos el caso de una mujer de 50 años en tratamiento inmunodepresor por trasplante renal en contexto de nefropatía diabética que se derivó a nuestro centro por derrame pleural izquierdo a estudio.

**Figura 1:**
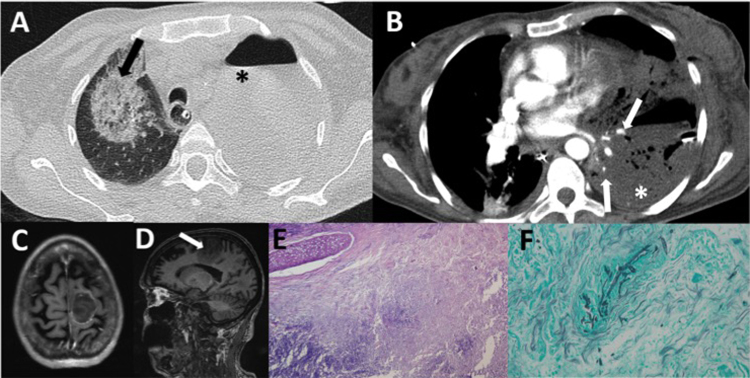
A) Corte axial de la TC torácica basal en ventana de pulmón, que muestra área en vidrio deslustrado con signo de halo invertido en pulmón derecho (flecha negra) e hidroneumotórax izquierdo (asterisco negro). B) Corte axial de la TC torácica con contraste intravenoso en ventana de partes blandas, que muestra áreas hipodensas sugestivas de infarto en segmentos basal anteromedial y lateral del lóbulo inferior izquierdo (asterisco blanco), con amputación proximal de las arterias segmentarias de las zonas afectas (flechas blancas). C y D**)** Corte axial de la RM T1 y sagital T1 que muestran lesión redondeada parasagital izquierda en forma de cuña (flechas blancas), sugestiva de corresponder a infarto venoso. E) Tinción de hematoxilina y eosina a 40 aumentos: parénquima pulmonar desestructurado, con extensas áreas de necrosis, *debris* celular e inflamación aguda. F**)** Tinción de plata metenamina a 200 aumentos: presencia de hifas en el interior de un vaso, con morfología compatible con mucor. Presencia en la periferia de levaduras compatibles con cándida.

A su llegada presentó cuadro de hemiparesia faciobraquiocrural derecha y disartria. Se realizaron una tomografía computarizada (TC) craneal y torácica. La TC torácica basal, que mostró hidroneumotórax izquierdo. El parénquima pulmonar se encontraba colapsado con una zona de cavitación en el lóbulo inferior izquierdo. En el pulmón derecho se observaron áreas en vidrio deslustrado con halo invertido sugestivas de infección fúngica de tipo aspergilosis^3^, ^4^ (fig. 1A). La TC craneal mostró una lesión hipodensa en parénquima frontal parasagital izquierdo (figs. 1C y D). Al cabo de 24 h se amplió el estudio con una RM cerebral, donde se observó el incremento de las dimensiones de la lesión y el edema citotóxico y vasogénico, sugiriendo infarto un cerebral de origen indeterminado.

En un control de la TC con contraste intravenoso realizado a los 4 días, persistía la gran consolidación del pulmón izquierdo con 2 áreas hipodensas sugestivas de infarto que afectaban a la língula y a los segmentos basal anteromedial y lateral del lóbulo inferior izquierdo. Se evidenció amputación proximal de las arterias segmentarias de las zonas afectas (fig. 1B). La afectación pulmonar derecha había progresado con nuevos focos de vidrio deslustrado. Ante estos hallazgos la orientación diagnóstica fue invasión fúngica que por su agresividad vascular sugirió mucormicosis^1^, ^2^.

Se realizó lobectomía inferior izquierda y lingulectomía^5^. La anatomía patológica confirmó que se trataba de una infección micótica tipo mucor, así como neumonía necrosante por micosis angioinvasiva (figs. 1E y F). La paciente también se sometió a neurocirugía por escisión de tumoración cerebral y la anatomía patológica mostró focos de necrosis asociados a cambios de vasculitis compatibles con embolia séptica fúngica.

Dada la elevada mortalidad de la enfermedad, la clave para el tratamiento es un diagnóstico precoz que permita la administración de anfotericina B liposomal y desbridamiento quirúrgico del tejido afecto^5^. Un buen conocimiento de los factores de riesgo y de las características por imagen es esencial para sugerir el diagnóstico.
