# Supplementary material for: Importance of Contrast-Enhanced CT in the Early Diagnosis of Pulmonary Mucormycosis: A Case Report
Source: Open Respir Arch. 2021 Mar 24;3(2):100091. [Article in Spanish] doi: 10.1016/j.opresp.2021.100091 (PMC10369601; doi:10.1016/j.opresp.2021.100091)
Supplement: Supplementary file 1 [file mmc1.docx]

**Figura 1. A:** Corte axial de TC torácica basal en ventana de pulmón que muestra área en vidrio deslustrado con signo de halo invertido en pulmón derecho (flecha negra) e hidroneumotórax izquierdo (asterisco negro). **B:** Corte axial de TC torácica con contraste intravenoso en ventana de partes blandas que muestra áreas hipodensas sugestivas de infarto en segmentos basal anteromedial y lateral del lóbulo inferior izquierdo (asterisco blanco) con amputación proximal de las arterias segmentarias de las zonas afectas (flechas blancas). **C y D:** Corte axial de RM T1 y sagital T1 que muestran lesión redondeada parasagital izquierda en forma de cuña (flechas blancas), sugestiva de corresponder a infarto venoso. **E:** Tinción de hematoxilina y eosina a 40 aumentos: parénquima pulmonar desestructurado, con extensas áreas de necrosis, debris celular e inflamación aguda. **F:** Tinción de plata metenamina a 200 aumentos: presencia de hifas en el interior de un vaso, con morfología compatible con mucor. Presencia en la periferia de levaduras compatibles con cándida.
